# Editorial: Current research on serological analyses of infectious diseases

**DOI:** 10.3389/fmed.2023.1154584

**Published:** 2023-02-17

**Authors:** Eric William Rogier, Emanuele Giorgi, Kevin Tetteh, Nuno Sepúlveda

**Affiliations:** ^1^Malaria Branch, Division of Parasitic Diseases and Malaria, Centers for Disease Control and Prevention, Atlanta, GA, United States; ^2^Centre for Health Informatics, Computing, and Statistics, Lancaster University, Lancaster, United Kingdom; ^3^Department of Infection Biology, Faculty of Infectious and Tropical Diseases, London School of Hygiene and Tropical Medicine, London, United Kingdom; ^4^Department of Mathematics & Information Science, Warsaw University of Technology, Warsaw, Poland; ^5^Centro de Estatística e Aplicações da Universidade de Lisboa (CEAUL), Lisbon, Portugal

**Keywords:** microarray, immunochromatography test, enzyme-linked immunosorbent assay (ELISA), multiplex bead array, neglected diseases, tropical diseases, SARS-CoV-2, disease diagnosis and analysis

## Introduction

Serology based on antibody detection or quantification is a key research tool in the analysis of human infectious diseases. In Public Health and Epidemiology, it allows the estimation of the disease burden beyond the classical measures based on the presence or frequency of active infections in the population (1, 2). It also allows the prediction of when individuals were previously infected for tailoring novel disease control strategies (3, 4). In Medicine, it can assist in diagnosis (5), in the inference of disease etiology and pathology (6–8), and in the stratification of patients for better disease management and treatment (9). All these research opportunities motivated a discussion about the creation of a World Serum bank for infectious diseases (10–12).

Until recently, the enzyme-linked immunoassay (ELISA) and other related tests were at the core of the research made in infectious diseases. These tests typically detect or quantify antibodies against a single antigen. Nowadays high-throughput serological technologies, such as microarrays and multiplex bead assays, are becoming competing rivals of these standard tests due to the possibility of measuring multiple antibodies in the same biological sample at a reasonable cost. As such, these new technologies are giving rise to multiple system serology analyses (13–16).

**Figure 1:**
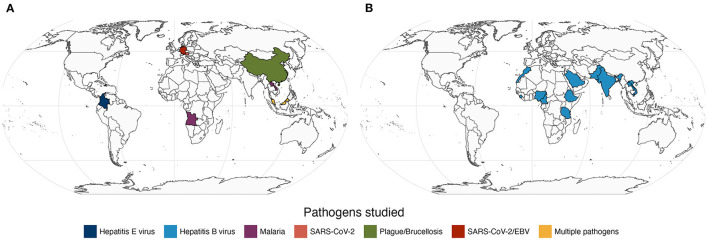
Map of the study populations and the respective pathogens featured in 9 original research papers **(A)** and in one systematic review **(B)** related to seroprevalence of the Hepatitis B virus among health workers (Maamor et al.). Note that Germany contributed with two independent papers (Girl et al. and Sepúlveda et al.) in plot A.

In this Research Topic, we took the pulse of current research of infectious diseases based on serology with a special focus on applications in Medicine, Epidemiology, and Public Health. Among more than 20 submissions received, we were able to collect 9 original Research Topics (Figure 1A) and one systematic review (Figure 1B). These papers featured diverse infectious diseases, including neglected, re-emerging, tropical, established and novel. We were pleased to have at least one study investigating populations from different continents. This suggests that the findings of this Research Topic are likely to benefit multiple human populations globally. Below we provide a brief context of the published studies.

## Serological studies using standard antibody assays

Notwithstanding the technological advances in serology, standard antibody assays seem the only viable option for investigating many diseases or populations from low- and middle-income countries (LMIC). This idea is illustrated in three independent studies included in the Research Topic. In one study, Qin et al. used serum agglutination tests to estimate the seroprevalence of animal plague and brucellosis in the neglected populations of Qinghai-Tibet plateau; these two neglected diseases are re-emerging in the area and, therefore, the reported findings have the potential to support more effective disease control interventions in the affected populations. In another study, Fernández Villaslobo et al. estimated the seroprevalence of Hepatitis E virus in children and adolescents in Colombia's capital city of Bogotá using commercial ELISA. Several study limitations were identified by the authors (e.g., massive school closure due to COVID-19 pandemic), but the findings suggested a low seroprevalence of IgG antibodies (~1%) and a single case of active infection (detected by IgM reactivity) among the study participants. It would be interesting to know whether these findings hold true if antibodies against multiple antigens were measured in the study. In a third study, Maamor et al. summarized data of 25 reports on the seroprevalence of Hepatitis B virus among health workers. These reports were restricted to African and Asian LMIC (Figure 1B) and, therefore, it is no surprise that the respective data were based on ELISA or rapid antibody tests whose performance can be affected by transport and storage conditions among other factors.

In the emergency of containing a disease outbreak, it is desirable to conduct a large-scale epidemiological study in real time. In this scenario, classical serological assays are invaluable tools in the field due to their low cost, the use of relatively simple technology, and the easiness of protocol's standardization across participating labs. The valuable use of these assays was illustrated in the peak of the COVID-19 pandemic with the fast execution of studies using ELISA protocols or rapid immunochromatography tests (17–19). In this scenario, it is fundamental to know the performance of available assays or tests in the field, as done in the United Kingdom and Denmark (20, 21). With a similar purpose of these benchmark studies, Girl et al. evaluated the performance of 2 lateral flow assays and 2 surrogate ELISA tests to detect SARS-CoV-2-neutralizing antibodies using data from more than 300 German individuals. Notwithstanding the current dampening of the COVID-19 threat in many parts of the world, this and similar studies provide a solid basis of evidence for facing future resurgences of SARS-CoV-2 in the respective populations.

## Serological studies using high-throughput antibody assays

High-throughput antibody technologies are becoming more popular among the scientific community. Our Research Topic captures somehow this increasing popularity given that more than half of the published studies featured the use of these avant-garde assays in different epidemiological contexts. Similar to a nationwide seroprevalence study from Spain (22), Willeit et al. illustrated the benefits of using a commercial high-throughput and automated immunoassay to screen rapidly antibodies against the spike protein of SARS-CoV-2 B1.351 variant in almost 2,500 individuals from an Austrian district. In Sepúlveda et al., more than 3,000 different antibody responses to the common Epstein-Barr virus were screened in German patients with Myalgic Encephalomyelitis/Chronic Fatigue Syndrome (ME/CFS) and healthy controls using a seroarray. The rationale for conducting a massive antibody screening lies on the fact that this complex and neglected disease remains without a diagnostic biomarker. This study highlighted two candidate antibody targets (EBNA4_0529 and EBNA6_0070) that could not only help in diagnosing a large subset of ME/CFS patients, but also further support the autoimmune hypothesis for the pathogenesis of this disease (23). In the realm of tropical diseases, Rogier et al. conducted a detailed analysis of multiple antibody responses to the malaria-causing *Plasmodium falciparum* in Angolan children using a multiplex bead assay. The respective findings provide additional evidence for the current understanding of malaria immunity. These and other findings from the literature set the foundation to innovate on malaria vaccine development and immune-related treatments. In another study of Malaria, Byrne et al. reported data from more than 5,000 individuals from the Lao People's Democratic Republic using a multiplex bead assay. This study illustrates how multiplex data can be used to construct risk maps that could detect important foci of infection for close surveillance and future interventions.

The most impressive applications of multiplex serological assays in this Research Topic are related to two studies where data allowed to investigate multiple diseases at the same time; other examples of studies based on similar ideas can be found elsewhere (24–26). In Chan et al., the study was performed in the Malaysian province of Sabah and contemplated the sampling of more than 10,000 individuals. The data comprised antibodies against twelve antigens related to 6 neglected tropical diseases, including lymphatic filariasis, yaws, and trachoma. In another study, Chan et al. extended the number of measured antibodies to investigate the epidemiology of 11 pathogens in Haiti. This study integrates a series of research efforts to support malaria elimination in the country (27–31). This study showed the benefit of using multiplex data to screen secondary diseases in a single timepoint, thus, avoiding the negative effects (e.g., increasing cost and participation fatigue) of conducting multiple sampling in the same population.

## Conclusion

This Research Topic provides an interesting contrast between studies using standard serological tools and those using more advanced technology. In our perspective, this contrast is particularly important to judge the pros and cons of using one or another technology across different research contexts. Above all, the availability of more advanced serological technology is a great opportunity to foster collaboration among researchers and to enhance capacity building (e.g., lab automation and data analysis skills) where it is needed the most.

In summary, this Research Topic shows the increasing popularity of high-throughput serological data and how these can be useful in the support of public health and epidemiological interventions (e.g., creating of multiple risk maps based on different antibodies to identify the key foci of infection). It also shows how the same type of data can expand our current knowledge on the pathogenesis and diagnosis not only of infectious disease but also of diseases with unknown etiology, as illustrated for ME/CFS.

## Author contributions

NS drafted the editorial. All authors revised and approved the final version of the editorial.
